# The Role of the Advanced Practice Provider in a Pericardial Center of Excellence

**DOI:** 10.1007/s11886-025-02205-y

**Published:** 2025-03-10

**Authors:** Mary Heine, Ankit Agrawal, Emma Wensink, Tom Kai Ming Wang, Allan Klein

**Affiliations:** 1 Cleveland Clinic, Cleveland, OH USA; 2 Center for the Diagnosis and Treatment of Pericardial Diseases, Section of Cardiovascular Imaging, Department of Cardiovascular Medicine, Heart, Vascular, and Thoracic Institute, Cleveland Clinic, Cleveland, Ohio USA

**Keywords:** Advanced practice provider, Pericardial center, IL-1 blocker, Recurrent pericarditis

## Abstract

**Purpose of Review:**

Pericarditis can be a chronic and complex disease requiring years of treatment. These patients require close monitoring of labs, medications and their side effects, as well as virtual and inpatient follow up. Due to the complexity of this disease, many of these patients are treated for months and sometimes years. Our review highlights the role Advanced practice providers (APP) play in managing the complexity of these patients by providing efficient and quality care.

**Recent Findings:**

Multi-modality cardiac imaging is the cornerstone to the evaluation and treatment of pericardial diseases. The addition of Interleukin (IL-1) blockers or biologics (Rilonacept, Anakinra) in the last few years provides targeted therapy for these patients. Using imaging guided therapy (IGT) these complex patients require close, continuous follow up and monitoring as well as frequent medication titration.

**Summary:**

The outcomes for these pericardial patients are improved with these centers due to the specialized medical and surgical care. Advanced Practice Providers play a vital role in a pericardial center with ordering the appropriate imaging and labs, handling medication titration, and providing patient education and continuity of care for these patients. They have been shown to decrease mortality, increase quality of care, and increase medication adherence.

## Introduction

### Pericarditis

Pericarditis is defined as inflammation of the pericardial sac containing the heart [1]. Pericarditis is a common cause of chest pain leading to numerous emergency room visits and hospitalizations each year [2]. It represents 5% of emergency room visits for nonischemic chest pain [3]. The etiology of this disease process is most often idiopathic or viral in developed countries, and Tuberculosis (TB) is the most common cause in developing countries. Other common causes include post cardiac injury due to post-cardiac/thoracic procedures as well as autoimmune diseases related to systemic lupus erythematosus, rheumatoid arthritis, scleroderma, inflammatory bowel disease, or Sjogren’s syndrome [4]. Less common etiologies include neoplasms, radiation, primary cardiac injury syndromes such as myocardial infarction, and congenital and metabolic disorders [4].

The types of pericarditis range from acute, chronic, incessant, recurrent, and constrictive. With the appropriate diagnosis and treatment 70–85% of patients will have a benign course and resolution of symptoms without recurrence when treated with first line therapy of non-steroidal anti-inflammatories (NSAIDS) and colchicine. Of patients with acute pericarditis, 15–30% can develop recurrent pericarditis, and half of these patients will have multiple recurrences [4]. A very small percentage of patients can develop constrictive pericarditis and, in rare instances, will require pericardiectomy [4]. In addition, transient constrictive pathophysiology should be quickly identified as it is a reversible form of constrictive pericarditis with ongoing inflammation without progression to chronic constriction. With early diagnosis, these patients can be treated with appropriate pharmacological methods to avoid surgery [4].

### Diagnosis and Evaluation

**Fig. 1:**
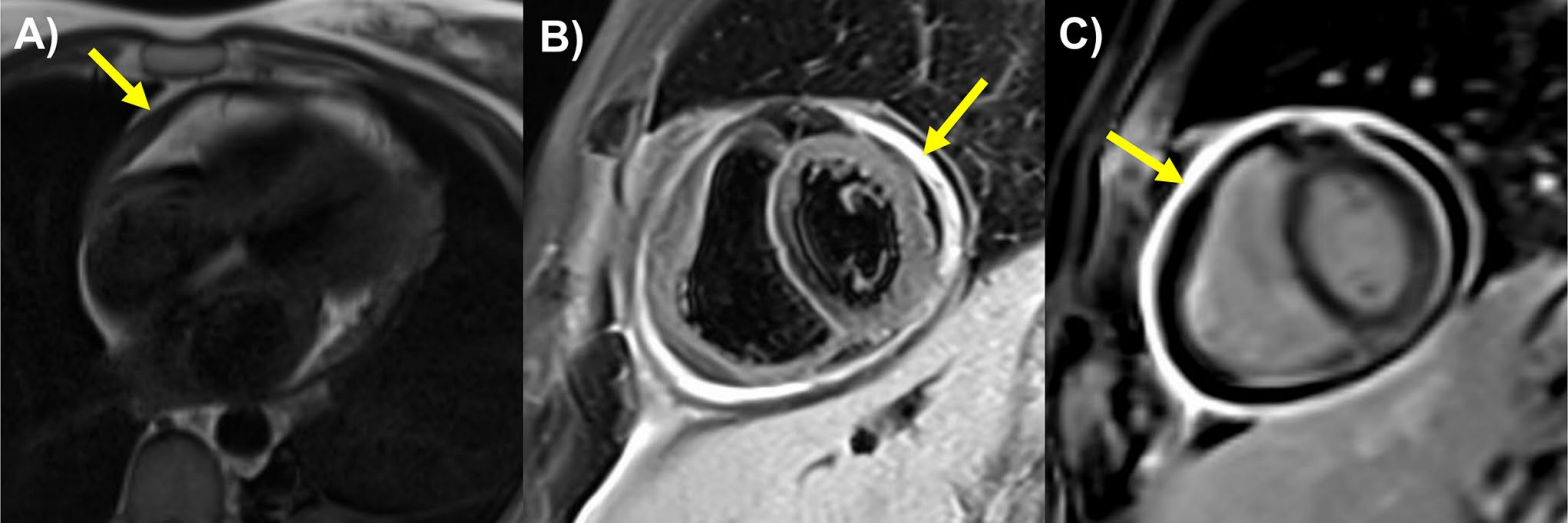
Cardiac magnetic resonance case of recurrent pericarditis (**A**) Black-blood spin echo sequence showing pericardial thickening (arrow); (**B**) T2-short tau inversion recovery sequence showing increased signal of the pericardium suggesting pericardial edema; (**C**) Phase-sensitive inversion recovery sequence showing pericardial late gadolinium enhancement suggesting neovascularization and inflammation

The 2015 European Society of Cardiology (ESC) guidelines for diagnosing pericarditis include two of the following four criteria. These criteria include pleuritic chest pain that is consistent with classical pericardial pain, which improves on leaning forward and worsens on laying back, pericardial friction rub, Electrocardiogram (ECG) showing diffuse ST-segment elevation or PR segment depression, and new or worsening pericardial effusion [5]. Initially, in most cases, these patients are diagnosed with ECG changes and elevated inflammatory markers such as C-reactive protein (CRP) and sedimentation rate (ESR) [5]. Multi-modality cardiac imaging has become a cornerstone in the evaluation, prognostication, surveillance, and assessing treatment response in pericardial diseases [6]. Echocardiography (Echo) remains the first line of imaging for pericardial evaluation due to its non-invasive assessment capability, availability, and low cost. It is primarily used for assessing pericardial effusion, including size and signs of tamponade and constriction. Computed tomography (CT) can provide supplementary information to assess pericardial effusion, calcifications in constriction, and fluid and tissue characterization of effusions and masses. Cardiac magnetic resonance (CMR) has been used more frequently to delineate and qualitatively grade pericardial inflammation on late gadolinium enhancement (LGE) and edema using T2 short-tau inversion recovery (T2-STIR) shown in Fig. 1.CMR can confirm pericarditis, predict, and provide imaging guided therapy (IGT) and monitor response or progression [7]. CMR is also useful for identifying constrictive physiology, pericardial effusions, and characterizing pericardial masses [6].

### Management

In recent years, there has been a paradigm shift in the management of pericarditis. Initial therapy remains a combination of colchicine and non-steroidal anti-inflammatories (NSAIDS) or aspirin, and exercise restriction which leads to a resolution in most cases. However, a minority of cases progress to incessant, recurrent, or chronic pericarditis [8, 9]. The traditional second-line treatment is corticosteroids; however, these have many side effects and are associated with recurrence when weaning, so they are no longer the best option [8]. Contemporary clinical trials AIRTRIP and RHAPSODY [10] have demonstrated the efficacy and safety of interleukin-1 (IL-1) blockers anakinra and Rilonacept, respectively as well as the goflikicept trial, in treating in the acute flare and reducing recurrence and improving symptoms and inflammatory markers. The current recommendations support earlier use of IL-1 blockers as second-line therapy, especially when patients have an inflammatory phenotype [11]. The use of IGT with CMR and early IL-1 blockers can be efficiently done at a pericardial center of excellence that has the capability of multi-modality imaging such as Echo, Cardiac MRI and CT as well as access to a pericardial specialist, cardiac surgery, rheumatology, and a specialty pharmacy as well as a dedicated nurse practitioner to manage these patients [8]. Treatment for these patients can last up to 5–7 years depending on the severity of the inflammation.

## The Role of the Advanced Practice Provider

### Managing a Chronic Disease

There are several studies illustrating the potential of nurse-led clinics for complex cardiovascular diseases. Nurse-led syncope, atrial fibrillation, and heart failure clinics show promise of improving patient’s management of symptoms [12]. They have been shown to decrease mortality, increase quality of care, and increase medication adherence [12]. They also reduce healthcare utilization and costs. The benefits that these clinics provide can be extrapolated to the role of the APP regarding pericardial disease clinics in tertiary centers, which provide care to a high volume of patients [13]. These patients also require frequent laboratory investigations, management, and tapering of medications and their side effects. There is also a need for education about this chronic disease and the medications used to manage it.

### Pericardial Center of Excellence

**Fig. 2:**
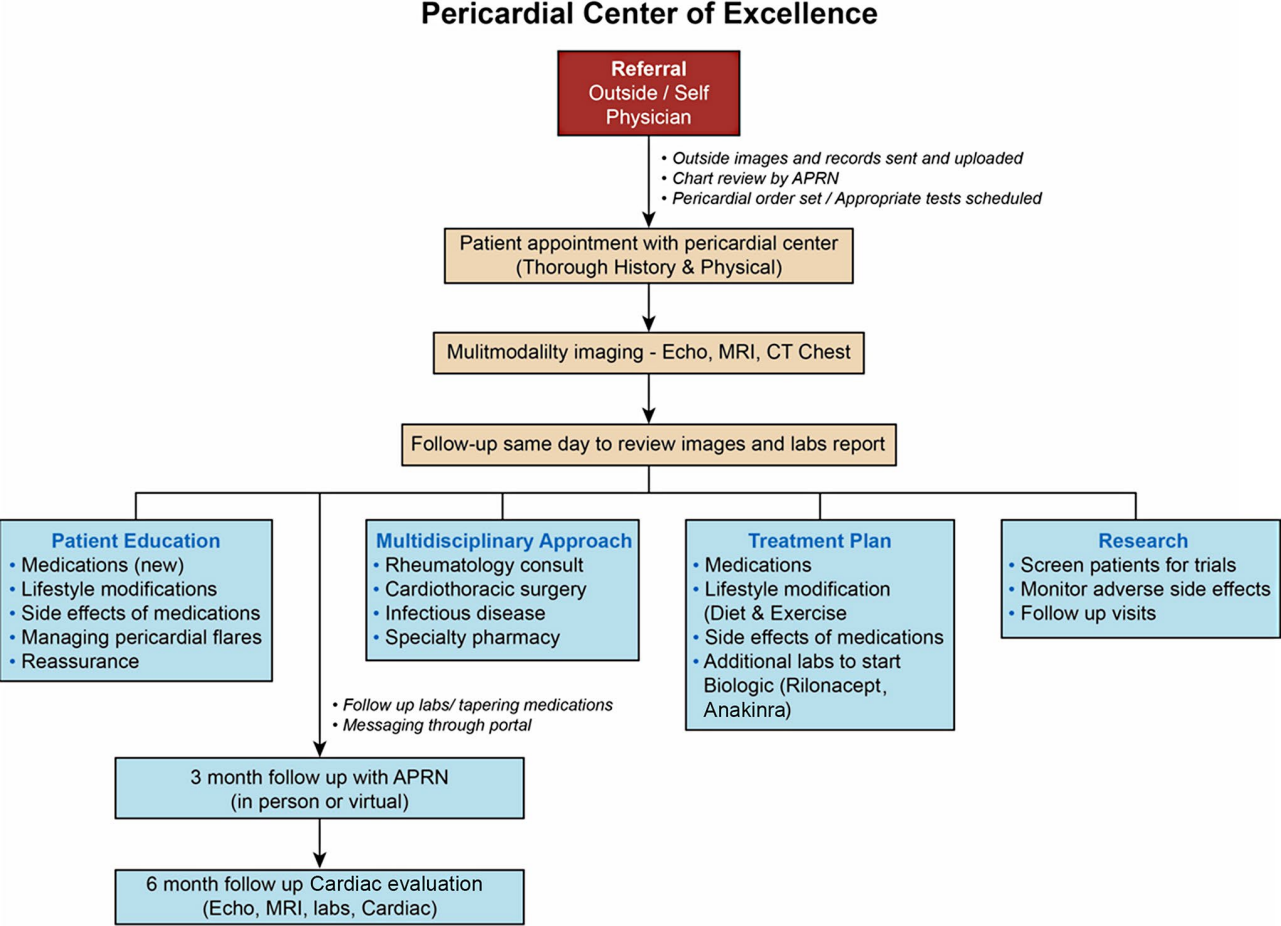
Flow Diagram of Pericardial Center of Excellence

A pericardial center of excellence is defined as a multidisciplinary center of excellence where a patient with pericarditis can see a pericardial specialist, receive or confirm a diagnosis, and see a multidisciplinary team over a span of two days. The flow of care in this center is shown in Fig. 2. They will have specialized imaging, including Cardiac MRI as well as Echo, ECG and labs during the first visit. Many of these patients travel significant distances to see a pericardial specialist and it is difficult for them to return often for appointments. Therefore, it is more effective to complete this process in 1–2 days. The APP plays a vital role in coordinating care, managing treatment, and providing education for these patients. The outcomes for these pericardial patients are improved significantly with these centers due to the specialized medical and surgical care. If necessary, they can see various specialty providers related to their diagnosis such as rheumatology, infectious disease, as well as consult with cardiothoracic surgery if necessary.

### Multidisciplinary Approach

The benefit of coming to a Pericardial Center of Excellence is having a multidisciplinary team evaluate the patient. This team consists of members from administration, clinical (physicians, APP, and nurses), and pharmacy. Ideally the center would have multiple pericardial specialists and APPs who can treat these patients. A pericardial center should have a minimum of one pericardial specialist and a designated APP and manage at least 100 patients with complex pericardial disease per year.

The patient can also be seen by other cardiac specialties, such as electrophysiology or heart failure, for optimization of medical therapy. Any further testing, such as right or left heart catheterization to assess LV filling pressures or stress testing, metabolic stress echocardiography if appropriate, is scheduled. They will also be referred to rheumatology if there is any concern for autoimmune disorders to guide proper management, as these patients may be refractory to traditional therapy. In addition, the patient can be seen by cardiothoracic surgery if a total radical pericardiectomy is in the imminent future.

### APP Qualifications and Function

In order to function in the pericardial center, the APP would require formal and informal qualifications. Formal qualifications include having a master’s degree in nursing or Master of Science in physician assistant studies. Informal qualifications include knowing what criteria is present to diagnose pericarditis including elevated inflammatory markers, ECG changes, and clinical signs and symptoms. They should also have a background in cardiology and the basic knowledge of the diagnostic imaging including Echo and CMR. They should have some experiencing providing care in the outpatients setting and seeing patients in a timely manner and ordering appropriate labs and testing as well as be familiar with the Electronic Medical Record (EMR) used in that institution. In addition, a training period of about six weeks where the APP can shadow a cardiologist and another APP and become familiar with various types of pericardial patients including those diagnosed with acute, chronic, and constrictive disease would be ideal. The APP should also have an experienced provider to direct specific questions to. At the end of the orientation period, they should be proficient in seeing a full schedule of nine to ten patients independently. Once the APP has been properly trained, they will follow established pericardial patients or patients that have already been seen at least once by the cardiologist. These appointments can be done in person or virtually if patients are coming from long distances to provide more longitudinal care, manage a larger volume, and retain patients. Virtual visits are especially effective when trying to manage medications and tapering for a large volume of patients. About 50-60% of their time would be devoted to patient care.

Ideally, the APP would have administrative and research time. They would devote this time to do chart review of new pericardial patients, answering messages in the patient portal, and following labs and test results. This time would also be devoted to screening incoming and established patients for clinical research studies and co-authoring manuscripts on pericardial disease as well as presenting at conferences. They will also be involved in educating other clinicians, APPs, and multi-disciplinary teams on these pericardial conditions.

### Referrals

The pericardial center receives referrals internally and externally from various services, the local emergency department, and all the domains of cardiology, cardiothoracic surgery, and rheumatology. There are also patients who are self-referred and make appointments though general scheduling. The timeline for patient assessment and management is dependent on acuity and determined on a case-by-case basis. There is a standard order set for these patients being seen in the pericardial clinic, including comprehensive lab work to include inflammatory markers, ECG, Echo, CT scan, and Cardiac MRI. Outside records and images are also collected and reviewed before the patient’s appointment. Due to the number of referrals, the patients are assigned to a pericardial specialist, and appointments are given based on acuity. The APP reviews each new patient chart to ensure all necessary testing and imaging are ordered. The APP then discusses each new patient and prior imaging with the cardiologist before the appointment. Any additional tests and consultations with various specialties, such as rheumatology, infectious disease, and cardiothoracic surgery, are scheduled prior to the appointment if necessary.

### Patient Evaluation and Plan of Care

The patient is first seen by a cardiologist specializing in pericardial disease, where a thorough history and physical are performed. This includes the date of onset, symptoms, number of emergency department visits and hospitalizations related to pericarditis, number of recurrences, prior testing and treatment, and past inflammatory markers. It is important to establish the pericarditis burden of the initial visit. The patient is next sent for the scheduled tests and results of all imaging and labs are reviewed with the patient the same day. If a biologic like rilonacept is presumed to be started, then additional investigations (tuberculosis screening, human immunodeficiency viral screening panel, Hepatitis B panel, and test for pregnancy) are done, and the paperwork for the medication is initiated. After testing is completed, the plan of care is then discussed with the patient. The APP is present for this discussion. If the medications they are on are escalated to higher doses, the nurse practitioner will review the tapering, set up laboratory work, and schedule the appropriate tests and follow-up appointments.

If any new medications such as biologics are recommended, the APP will provide information about this drug, its side effects, how to manage them, and the duration of therapy. They will also set up recurring laboratory investigations such as C-reactive protein (CRP), High sensitivity CRP (HS-CRP), review the tapering schedules, and once again set up appropriate follow-up and place these orders. Once a diagnosis of pericarditis is made, the patient must also make lifestyle modifications. Exercise, activity, and heart rate restrictions, along with diet recommendations, are also part of this discussion. It is important for the patient to limit their activity, keeping heart rate less than 100 bpm until symptoms have fully resolved and inflammatory markers have normalized and if pertinent CMR shows near resolution of inflammation. Even with these restrictions patients can have a flare up with resuming intense physical activity [15].

### Patient Education and Support

Initiating any new medication, especially IL-blockers, requires patient education. The patients often need assistance with filling out the paperwork and understanding the process for obtaining the medication and the cost. The APP works closely with specialty pharmacies for insurance authorization and communicates with the patient during this process. The APP also functions as liaison between the patient and pharmaceutical company to help access patient support programs for financial assistance and education. Recurrent laboratory orders are also initiated as these patients will be on an aggressive tapering schedule for the steroids, nonsteroidal, and anti-inflammatories once the biologic is started. Once on the biologic for one-month, inflammatory markers are checked every two weeks until the patient is on single therapy. They also have a Complete Blood Count (CBC) and Complete Metabolic Panel (CMP) every three months while on Rilonacept to monitor the white blood cell count (WBC) and liver function tests (LFTs). The lipid panel is also checked, as Rilonacept can elevate the triglyceride level over time. Any patient initiated on Rilonacept has a three-month check-in with the nurse practitioner, either in person or virtually. The three-month follow-up with the APP provides any further education and questions and reviews recent labs and any symptoms or side effects the patient may be experiencing. The six-month follow-up with the pericardial specialist includes follow-up imaging to monitor disease progression and improvement. It is often the case that peer to peer evaluations are periodically required to continue therapy and provide a rationale for follow-up imaging. The APP is responsible for these reviews and drafting appeal letters.

The patient also follows closely with the APP for adverse side effects of medications, pericardial flares, and escalation of therapy. These aid in avoiding emergency room visits or placing patients back on steroid therapy. The electronic medical record (EMR) is followed closely to monitor labs and is used as an essential mode of patient communication. The patient messages through the portal with any medication issues or symptoms. The EMR and patient portal are frequently used as patient messages for minor symptoms, medication management, and direction on the tapering schedule.

The complexity and chronicity of pericardial disease not only affects the physical health but also the mental health and quality of life of the patients. Patients with recurrent pericarditis report reduced levels of physical and mental health [14]. They are often misdiagnosed and or under-treated for many years and suffer from severe disease morbidity due to the unpredictability of the disease. They must refrain from physical activity and must restrict their activity for prolonged periods of time [15]. They also carry a fear of a recurrence of symptoms as they taper and wean off the medications. The practitioner’s role involves providing reassurance, optimal support, and resources for these patients who struggle with their mental health.

### Research

In addition, the APP assists with recruiting for clinical trials as well as updating the pericardial registries and works closely with primary investigators, research nurses, research fellows and coordinators. They screen patients for potential studies and assist with the patients being seen based on the study visits and follow-up required. Ideally, the patients are screened prior to their visit to assess their eligibility. They also monitor labs, imaging studies, and any adverse side effects associated with the trial and provide education about the clinical trial to eligible patients.

## Conclusions

The APP in a pericardial center of excellence has a multifaceted role. This is largely due to the recent use of cardiac MRI to quantify pericardial inflammation, guide treatment, and increase the use of IL- blockers. The role of the APP is necessary to manage these complex patients. It involves working with a multidisciplinary team to properly diagnose and treat the patients. It also requires close follow-up with the patient regarding symptoms, tapering medications, and managing their side effects. The APP provides not only efficient care for this complex patient population but also mental support due to the disease’s morbidity.

### Key References

- King-Dailey K, Frazier S, Bressler S, King-Wilson J. The Role of Nurse Practitioners in the Management of Heart Failure Patients and Programs. Curr Cardiol Rep. 2022;24:1945–56.
-
  - The potential of nurse-led clinics for complex cardiovascular diseases.
- The Paradigm Shift in the Management of Recurrent Pericarditis [Internet]. American College of Cardiology. [cited 2024 Sep 6]. Available from: https://www.acc.org/Latest-in-Cardiology/Articles/2022/12/19/14/52/http26;3a26;2f26;2fwww.acc.org26;2fLatest-in-Cardiology26;2fArticles26;2f202226;2f12&2f1926;2f1426;2f5226;2fThe-Paradigm-Shift-in-the-Management-of-Recurrent-Pericarditis.
-
  - The use of IGT with CMR and early IL-1 blockers can be efficiently done at a pericardial center.

## Data Availability

No datasets were generated or analysed during the current study.

## References

[1] Rehman I, Nassereddin A, Rehman A, Anatomy, Thorax. Pericardium. StatPearls [Internet]. Treasure Island (FL): StatPearls Publishing; 2024 [cited 2024 Sep 6]. Available from: http://www.ncbi.nlm.nih.gov/books/NBK482256/29489245

[2] The Role of the Physician Assistant and Nurse Practitioner in the Management of Recurrent Pericarditis at a Model Center for Pericardial Diseases [Internet]. American College of Cardiology. [cited 2024 Sep 6]. Available from: https://www.acc.org/latest-in-cardiology/articles/2016/06/22/11/43/http%3a%2f%2fwww.acc.org%2flatest-in-cardiology%2farticles%2f2016%2f06%2f22%2f11%2f43%2fthe-role-of-the-pa-and-np-in-the-management-of-recurrent-pericarditis

[3] Yesilyaprak A, Kumar AK, Agrawal A, Furqan MM, Verma BR, Syed AB, et al. Predicting long-term clinical outcomes of patients with recurrent pericarditis. J Am Coll Cardiol. 2024;84:1193–204. 39217549 10.1016/j.jacc.2024.05.072

[4] Al -Kazaz, Mohamed, Klein AL, Oh JK, Crestanello JA, Cremer PC, Tong MZ et al. Pericardial Diseases and Best Practices for Pericardiectomy. Journal of the American College of Cardiology [Internet]. 2024 [cited 2024 Sep 6];84:561–80. Available from: https://www.jacc.org/doi/10.1016/j.jacc.2024.05.048 10.1016/j.jacc.2024.05.048 39084831

[5] Adler Y, Charron P, Imazio M, Badano L, Barón-Esquivias G, Bogaert J, et al. 2015 ESC guidelines for the diagnosis and management of pericardial diseases: the task force for the diagnosis and management of pericardial diseases of the European society of cardiology (ESC) endorsed by: the European association for cardio-thoracic surgery (EACTS). Eur Heart J. 2015;36:2921–64. 26320112 10.1093/eurheartj/ehv318 PMC7539677

[6] Klein AL, Abbara S, Agler DA, Appleton CP, Asher CR, Hoit B, et al. American Society of Echocardiography clinical recommendations for multimodality cardiovascular imaging of patients with pericardial disease: endorsed by the Society for Cardiovascular Magnetic Resonance and Society of Cardiovascular Computed Tomography. J Am Soc Echocardiogr. 2013;26:965–e101215. 23998693 10.1016/j.echo.2013.06.023

[7] Cremer PC, Klein AL, Imazio M, Diagnosis R, Stratification. and treatment of pericarditis: a review. JAMA [Internet]. 2024 [cited 2024 Sep 20]; Available from: 10.1001/jama.2024.12935 10.1001/jama.2024.12935 39235771

[8] The Paradigm Shift in the Management of Recurrent Pericarditis [Internet]. American College of Cardiology. [cited 2024 Sep 6]. Available from: https://www.acc.org/Latest-in-Cardiology/Articles/2022/12/19/14/52/http%3a%2f%2fwww.acc.org%2fLatest-in-Cardiology%2fArticles%2f2022%2f12%2f19%2f14%2f52%2fThe-Paradigm-Shift-in-the-Management-of-Recurrent-Pericarditis

[9] Kumar S, Khubber S, Reyaldeen R, Agrawal A, Cremer PC, Imazio M et al. Advances in Imaging and Targeted Therapies for Recurrent Pericarditis: A Review. JAMA Cardiology [Internet]. 2022 [cited 2024 Sep 8];7:975–85. Available from: 10.1001/jamacardio.2022.2584 10.1001/jamacardio.2022.2584 35976625

[10] Klein AL, Wang TKM, Cremer PC, Abbate A, Adler Y, Asher C, et al. Pericardial diseases: international position Statement on New concepts and advances in Multimodality Cardiac Imaging. JACC Cardiovasc Imaging. 2024;17:937–88. 39111992 10.1016/j.jcmg.2024.04.010 PMC12156183

[11] Klein AL, Imazio M, Cremer P, Brucato A, Abbate A, Fang F et al. Phase 3 Trial of Interleukin-1 Trap Rilonacept in Recurrent Pericarditis. New England Journal of Medicine [Internet]. 2021 [cited 2024 Sep 6];384:31–41. Available from: https://www.nejm.org/doi/full/10.1056/NEJMoa2027892 10.1056/NEJMoa2027892 33200890

[12] King-Dailey K, Frazier S, Bressler S, King-Wilson J. The role of nurse practitioners in the management of heart failure patients and programs. Curr Cardiol Rep. 2022;24:1945–56. 36434405 10.1007/s11886-022-01796-0 PMC9702908

[13] Aldajani A, Bérubé M, Mardigyan V. How and why to set up a Pericardial Disease Clinic. Can J Cardiol. 2023;39:1149–51. 37172644 10.1016/j.cjca.2023.04.028

[14] Agrawal A, Kumar A, Yesilyaprak A, Zumpano J, Pozuelo L, Klein A. Quality of life in patients with recurrent Pericarditis. Can J Cardiol. 2024;S0828–282X(24):00409–4. 10.1016/j.cjca.2024.05.018 38810775

[15] Klein BM, Dugan ES, LaCombe AD, Ruthmann NP, Roselli EE, Klein AL et al. Complex Management Decisions in a Professional Athlete With Recurrent Pericarditis. JACC Case Rep [Internet]. 2022 [cited 2024 Sep 20];4:1090–3. Available from: https://www.ncbi.nlm.nih.gov/pmc/articles/PMC9481902/10.1016/j.jaccas.2022.05.015 PMC9481902 36124145

